# Transition for adolescents with a rare disease: results of a nationwide German project

**DOI:** 10.1186/s13023-023-02698-2

**Published:** 2023-04-25

**Authors:** Corinna Grasemann, Jakob Höppner, Peter Burgard, Michael M. Schündeln, Nora Matar, Gabriele Müller, Heiko Krude, Reinhard Berner, Min Ae Lee-Kirsch, Fabian Hauck, Kerstin Wainwright, Sylvana Baumgarten, Janet Atinga, Jens J. Bauer, Eva Manka, Julia Körholz, Cordula Kiewert, André Heinen, Tanita Kretschmer, Tobias Kurth, Janna Mittnacht, Christoph Schramm, Christoph Klein, Holm Graessner, Olaf Hiort, Ania C. Muntau, Annette Grüters, Georg F. Hoffmann, Daniela Choukair

**Affiliations:** 1 Division of Rare Diseases, Department of Paediatrics, St. Josef-Hospital Bochum, Ruhr-University Bochum, Alexandrinenstraße 5, 44791 Bochum, Germany; 2 Centre for Rare Diseases Ruhr CeSER, Ruhr-University Bochum and Witten/Herdecke University, Bochum, Germany; 3 Centre for Child and Adolescent Medicine and and Centre for Rare Diseases, University Hospital Heidelberg, Heidelberg, Germany; 4 Department of Paediatrics III, University Hospital Essen, University Duisburg-Essen, Essen, Germany; 5 Centre for Evidence-Based Healthcare, Carl Gustav Carus Faculty of Medicine, University Hospital Carl Gustav Carus, Technische Universität Dresden, Dresden, Germany; 6 Institute for Experimental Paediatric Endocrinology and Centre for Rare Diseases, Charité - Universitätsmedizin Berlin, Berlin, Germany; 7 Children’s Department and University Centre for Rare Diseases (USE), University Hospital Dresden, Dresden, Germany; 8 Dr von Hauner Children’s Hospital, University Hospital and Munich Centre for Rare Diseases (M-ZSELMU), Ludwig-Maximilians-University Munich, Munich, Germany; 9 Institute of Public Health, Charité - Universitätsmedizin Berlin, Berlin, Germany; 10 Department of Paediatrics II and Essener Centre for Rare Diseases, University Hospital Essen, University Duisburg-Essen, Essen, Germany; 11 Martin Zeitz Centre for Rare Diseases, University Medical Centre Hamburg-Eppendorf, Hamburg, Germany; 12 Institute for Medical Genetics and Applied Genomics, University of Tuebingen, Tübingen, Germany; 13 Departments of Paediatrics, University Medical Centre Schleswig-Holstein, Campus Lübeck, Lübeck, Germany; 14 University Children’s Hospital, University Medical Center Hamburg Eppendorf, Hamburg, Germany

**Keywords:** Transition, Rare disease, Pathway, Empowerment, Health literacy, Adolescent health

## Abstract

**Purpose:**

The transition process from paediatric/adolescent to adult medical care settings is of utmost importance for the future health of adolescents with chronic diseases and poses even more difficulties in the context of rare diseases (RDs). Paediatric care teams are challenged to deliver adolescent-appropriate information and structures. Here we present a structured transition pathway which is patient-focused and adoptable for different RDs.

**Methods:**

The transition pathway for adolescents 16 years and older was developed and implemented as part of a multi-centre study in 10 university hospitals in Germany. Key elements of the pathway included: assessment of patients’ disease-related knowledge and needs, training/educational and counselling sessions, a structured epicrisis and a transfer appointment jointly with the paediatric and adult specialist. Specific care coordinators from the participating university hospitals were in charge of organization and coordination of the transition process.

**Results:**

Of a total of 292 patients, 286 completed the pathway. Deficits in disease-specific knowledge were present in more than 90% of participants. A need for genetic or socio-legal counselling was indicated by > 60%. A mean of 2.1 training sessions per patient were provided over a period of almost 1 year, followed by the transfer to adult care in 267 cases. Twelve patients remained in paediatric care as no adult health care specialist could be identified. Targeted training and counselling resulted in improved disease-specific knowledge and contributed to empowering of patients.

**Conclusion:**

The described transition pathway succeeds to improve health literacy in adolescents with RDs and can be implemented by paediatric care teams in any RD specialty. Patient empowerment was mainly achieved by individualized training and counselling.

**Supplementary Information:**

The online version contains supplementary material available at 10.1186/s13023-023-02698-2.

## Background

Adolescents with a chronic health condition are faced with the challenge to transition from paediatric to adult care (henceforth transition) systems within a short and defined period in their lives [1, 2]. Transition is a process for which the young patients need both to prepare and to be prepared for [3, 4].

Ideally, the transition period allows the adolescent to gradually take over the responsibilities as an adult patient. This includes adapting to a different care context and leaving familiar paediatric care teams who may have provided care for the patient from the beginning of life or the onset of illness. To enable the adolescent to complete this entire process, a successful transition requires careful planning and stepwise implementation [4].

According to a national survey in Germany about 15% of people under the age of 18 years are affected by a chronic disease with special care needs and will require a structured transition process [5]. However, transfer to a specialised adult care unit is often not accomplished [4] and in at least 40% of cases, the cessation of paediatric care marks a gap in the medical care with adverse consequences for therapy adherence, symptom control and the development of secondary diseases or even the development of irreversible damage [2, 6–9].

To address the above-mentioned challenges, transition pathways have been designed and implemented worldwide, mostly at local levels and often with a focus on a specific disease or group of diseases only. Unfortunately, these efforts frequently lack funding in the national health care systems and thus are not sustainable and cannot be implemented on a broader level [10, 11].

﻿Developing a transition process for adolescents with a rare disease (RD) is even more challenging for two main reasons: a) the difficulty in identifying experts/clinics in adult care and b) the necessity to provide disease-specific information to the patients. Rare diseases are a heterogeneous group of an estimated 5000–8000 different, mostly hereditary conditions and often manifest in childhood [12]. Paediatric medical care for these patients is mostly organized in tertiary care centres with highly specialised, multi- and interdisciplinary care teams working in in- and outpatient settings, to ensure close communication with the primary care paediatrician.

Over the last decades, a better understanding of the pathophysiological framework and improved treatment options have led to an increased and better survival of children with RDs and adolescents are transitioning at an increasing rate from paediatric into adult health care services. The latter are often not adequately equipped to meet the patients’ needs.

Previous studies have shown that encouraging young people to develop independence, both from their families and from their health care providers, will help to bridge the gap from paediatric to adult care and will encourage patients to make informed decisions about their wellbeing/care [13–15]. In addition, adolescents who are knowledgeable about their disease or condition show a deeper understanding of the implications of transitioning to adult care. Furthermore, patients who were able to explain their diagnosis in both lay and medical terms appeared to be more confident and communicated directly with providers instead of using their parents as a proxy and were self-assured in their ability to take care of themselves’ [14, 16–18].

Thus, to successfully transfer adolescents with rare and ultra-rare diseases into adult care, patients need to be trained and counselled intensively to become “experts on their own disease”. A structured transition pathway can achieve this result.

Here we describe the transition process and report the evaluation results of a multi-centre structured transition pathway for patients with rare diseases in Germany (TRANSLATE NAMSE).

## Material and methods

### TRANSLATE NAMSE

A German nationwide health care project ‘TRANSLATE NAMSE’, funded by the innovation fund of the Federal Joint Committee (G-BA, funding number 01NVF16024) was launched in April 2017, aiming to develop and implement patient pathways and care structures in 10 centres for rare diseases located at university hospitals. The study was approved by the ethics committee of the Charité—Universitätsmedizin Berlin (#EA2/140/17) and the local ethics committees [10, 19–21]

A structured transition and transfer pathway from paediatric to adult care for adolescents and young adults with RDs was developed and implemented in the context of this project [19]. Patients were recruited from December 2017 until January 2020.

### Patient pathway transition

The main goal of the transition pathway in TRANSLATE NAMSE was the quality-assured transfer of information from the paediatric treatment team to the adolescent patient, as well as to the health care provider(s) of the adult care team.

Adolescent patients with a RD from 5 participating hospitals (Heidelberg, Essen, Bochum, Dresden, and Hamburg), who were 15 years and older were invited to participate in the study during clinic visits by their paediatric specialists. Informed consent was obtained from patients and caregivers.

**Fig. 1:**
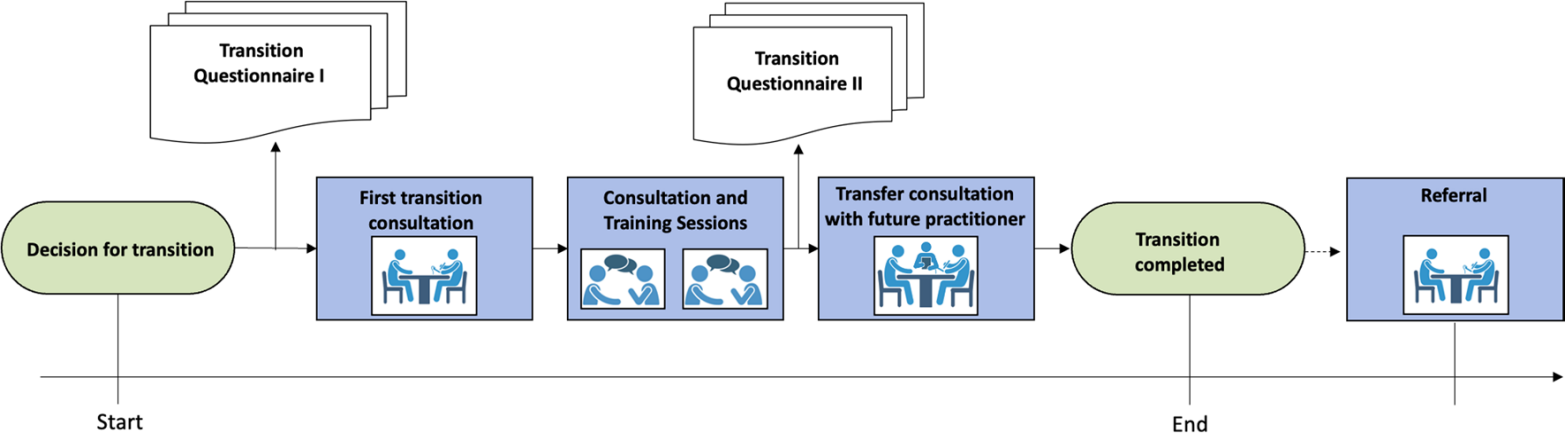
**Transition Pathway**: The transition process is initiated by the paediatric specialist., The first transition questionnaire is administered to the patients and (repetitive) training is offered according to the indicated needs. A structured epicrisis is prepared by the paediatric specialist. After the training sessions, the transition questionnaire is administered a second time to the patient to identify remaining gaps in health literacy. The transition pathway is completed with a transfer consultation that includes the explanation and hand-over of the epicrisis along with all accompanying documents as well as the scheduling of a follow-up appointment with the adult specialist

The pathway is described in detail [19, 22] in German only, and is therefore briefly summarized here and in Fig. 1 and Additional file 1: Fig. S1.

The transition process starts with an assessment of the patient’s disease-specific knowledge and need for counselling using a questionnaire that was developed for this project (Additional file 1: Fig. 2). The questionnaire was given to patients at the rare disease centre of the hospital participating in this project. The questionnaire consists of 30 items, such as disease-specific knowledge pertaining to diagnosis, therapy, monitoring and behavior management. Non-disease-specific items address the perceived level of independence when navigating the medical system and the need for genetic, legal and psychological counselling as well as career options. Quantification of the presently existing level of information and resulting training and consultation requirements was carried out using an excel-based evaluation matrix (Additional file 1: Table S1).

**Fig. 2:**
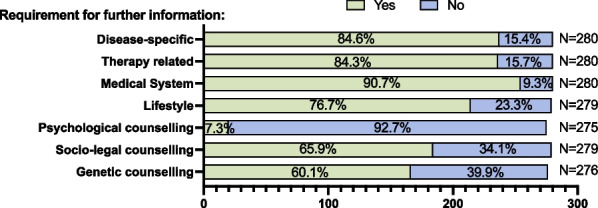
**Assessment of disease-specific knowledge** and indicated need for further information and counselling (green part of bar) in participating patients (number and percent)

Based on the assessment, training on health literacy was provided to patients by the paediatric treatment team of the centres for RD at the participating university hospitals in the areas with identified knowledge gaps during the follow-up consultation(s). Additional appointments regarding psychological, psychosocial, and/or human genetic topics were provided as indicated by the patient or if necessary.

A structured epicrisis including the relevant information from paediatric care and recommendations for further treatment and care was prepared by the paediatric specialist in charge. This information was explained and handed over to the patient no later than the transfer appointment.

After completion of the counselling and training sessions, the transition questionnaire was used to re-assess the level of knowledge and identify any remaining open questions. In addition, an appointment for the actual transfer of the patient was scheduled by the patient coordinator (case manager) from the centre for RD at the university hospital. This transfer appointment was preferably booked with the paediatric and the adult care physician at the adult clinic. During the transfer appointment the following items were addressed: the introduction of the new health care providers, the exchange and transfer of all relevant patient information, including genetic results, the explanation of the care process in adult medicine, and the delivery of the written epicrisis to the patient. The pathway was completed once a follow-up appointment was scheduled.

The beginning, duration and completeness of the patient pathway, the quantified level of disease-specific knowledge at two time points and the delivered training and counselling were documented in a checklist.

### Data collection and statistical analysis

Patient data were recorded in the participating centres in portable document format (PDF) forms, which were read as comma-separated values (CSV) files and pseudonymized merged across the centres. These were checked for plausibility and accuracy and analyzed using IBM SPSS Statistics 25 (SPSS Inc., Chicago, IL, USA) and R (R Development Core Team; 2019) programs. Data analysis for this process evaluation was descriptive: measures of central tendency are reported as mean and standard deviation or median and range (min–max). Inference statistics were not used, as the project’s objective was to provide information on a new care approach under real-world conditions.

Data from the CSV files were cross-checked with original data from the participating centres and additional information (e.g. on the diagnosis) was taken into account if applicable. As a result, case numbers in this report differ marginally from the official project report [22]. A calculation of the consumption of time- and financial resources in this pathway has been performed and published separately [10].

The evaluation of the TRANSLATE NAMSE project from the patients' point of view was carried out by means of questionnaires sent by mail. Participation in the evaluation was voluntary. No reminder letters were sent. The questionnaires were returned by mail by the patients to the evaluator centre for Evidence-Based Healthcare at the Technische Universität Dresden (Additional file 1: Fig. 3).

**Fig. 3:**
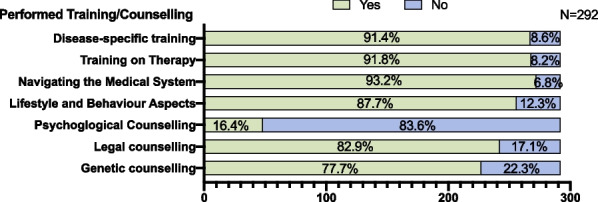
**Amount of training and counselling session** received by the participating patients (number and percent). Providing information by the specialists to the patients took on average 38.7 min (SD 23.2; median 37.5 min, range 5–130) for disease-specific information and 38.5 min (SD 29.5; median 30.0 min, range 5–230) for information on therapy. Genetic re-counselling averaged 18.1 min (SD 8.5; median 15 min; range 5–50) and psychological counselling took 98.7 min on average (SD 101.1; median 70 min, range 5–310)

## Results

### Patients’ characteristics

In total, 292 patients (146 male, 50.0%) were enrolled in the transition pathway. The mean age at inclusion was 18.0 ± 2.1 years. RDs were categorized into the following disease-specific groups: anaemia/primary immunodeficiencies (n = 11), autoinflammatory conditions (n = 60), endocrine conditions (n = 147), metabolic conditions (n = 16), nephrological conditions (n = 50) and other RDs (n = 8) (see Table 1). For diagnoses in these groups please refer to Additional file 1: Table S2.

**Table 1:** Patient’s characteristics

Condition	Haematologic/primary immune-deficiencies	Endocrine	Inflammatory	Metabolic	Nephrological	Other rare disease	Total
N	6	147	58	15	52	14	292
*Sex*							
Male	1 (16.6%)	78 (53.1%)	16 (27.6%)	6 (40.0%)	37 (71.2%)	8 (57.1%)	146 (50%)
Female	5 (83.3%)	69 (46.9%)	42 (72.4%)	9 (60.0%)	15 (28.8%)	6 (42.9%)	146 (50%)
*Age (years) at decision for transition*							
Mean	17.3	18.1	17.2	19.5	18.2	18.4	18.5
SD	1.4	2.0	1.3	4.4	1.8	3.4	2.1
Median	17.5	18.0	17.0	19.5	18.0	18.0	17.9
Min	15	15	16	15	16	16	15
Max	19	24	23	31	23	29	31

*Min* Minimum, *Max* Maximum, *N* Number, *SD* Standard deviation

Other Rare Diseases included neurologic, orthopaedic, gastrointestinal, and neoplastic diseases

### Training and counselling

#### Assessment of knowledge

Disease-specific knowledge gaps resulting in patient-centered education and counselling was assessed in 280 patients using the above-described transition questionnaire. In general, knowledge gaps (as indicated by answers: ‘Partially agree; ‘Disagree’) were present in many different areas for most patients at the beginning of the transition process (baseline t_0_) as displayed in Figs. 2 and 3. Knowledge gaps were most frequent regarding disease-specific aspects (need for education 84.6%) and regarding the ability to navigate the medical system independently (90.7%).

There was also a high need (65.9%) for socio-legal and genetic (60.1%) counselling, but few requests for psychological counselling (7.3%). The quantitative demand was evenly distributed among patients of the different disease groups.

#### Improving disease-specific knowledge

On average, 2.1 educational or counselling sessions and a total of 5.4 consultations or trainings were carried out per patient. Counselling was tailored according to the detected needs as displayed in Fig. 2. Counselling on how to navigate the medical system (independence), was received by 93.2% of patients, closely followed by counselling regarding disease-specific therapy (91.8%), on the disease itself (91.4%) and on lifestyle and behaviour aspects (87.7%). Legal (82.9%) and genetic aspects (77.7%) were counselled frequently, whereas psychological counselling was least frequent (16.4%) (Fig. 3).

#### Reassessment of health literacy and knowledge gaps

**Fig. 4:**
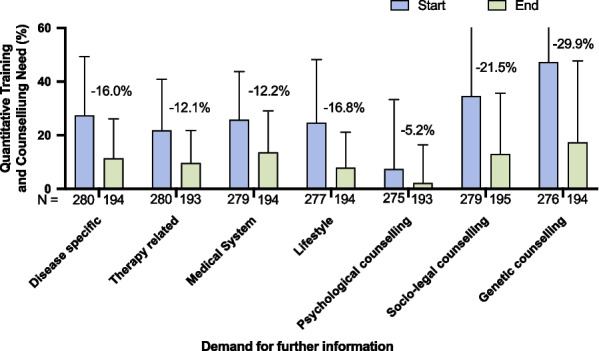
**Demand for information and/or counselling at the start and the end of the transition pathway.** Health literacy was assessed using the same questionnaire (Additional file 1: Fig. S2) at the beginning of the transition pathway (purple bars) and after training and counselling sessions (green bars). Quantitate need was assessed using the matrix from supp. Tab. 2

Reassessment of health literacy at the end of the patient pathway revealed significantly diminished requirements for information (Fig. 4). However, 78.5% of the patients still indicated a need for further advice regarding navigating the health care system.

#### Evaluation of the TRANSLATE NAMSE project from the patients’ point of view

One hundred and twenty-one patients (41.4%) participated in the final evaluation. Of those, 69.4% found the transition questionnaire helpful (40.4%, n = 49) or very helpful (28.9%, n = 35). Only 8 patients (6.6%) found it not helpful or not very helpful. On a scale of 1 (very good) to 5 (deficient), the entire transition pathway was rated 2.0 by the participants (SD 0.9; median 2, range 1–5).

### Transfer to adult care

**Fig. 5:**
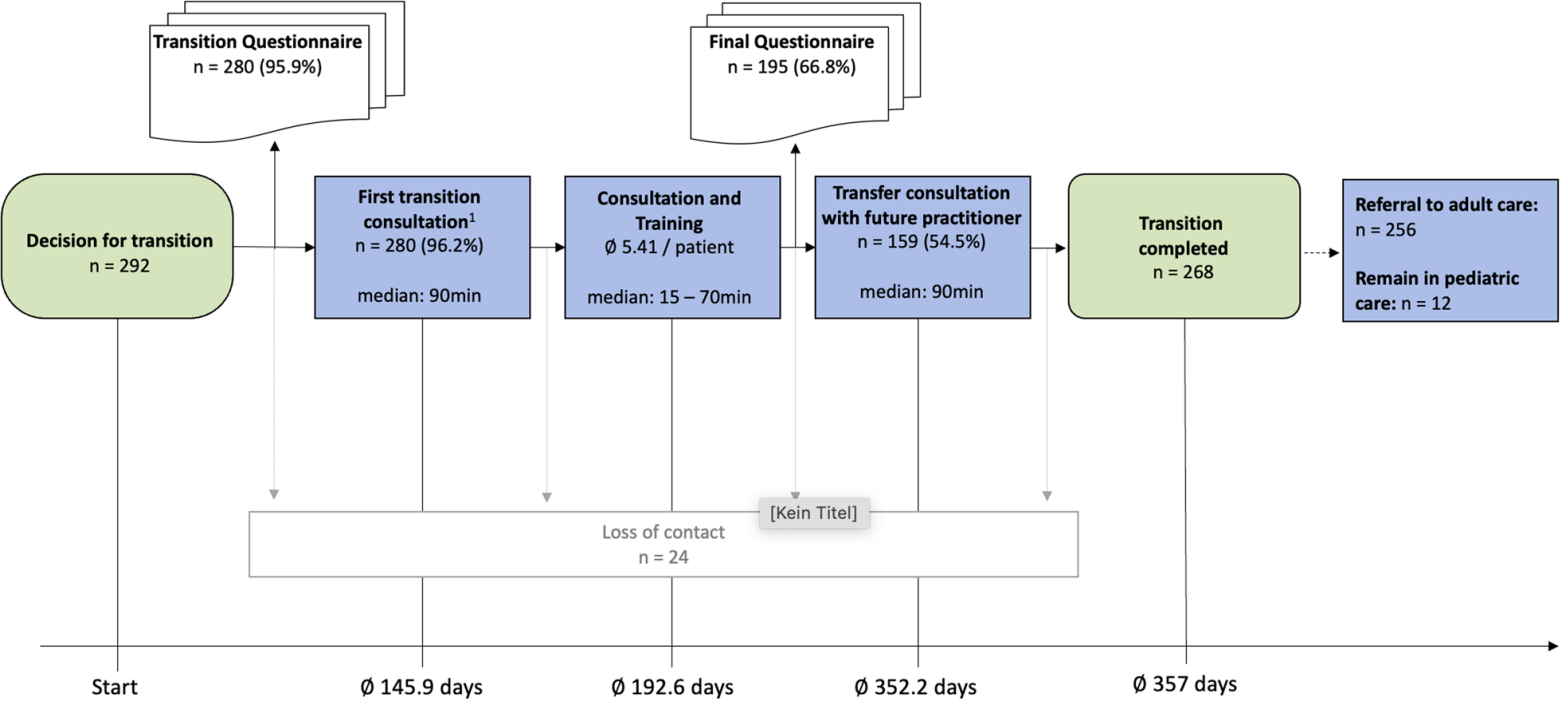
**Graphical display of the patient numbers** in the transition pathway. Abbreviations: min, minute; n, number; ∅, mean

Of the 292 patients who were initially included, 268 (91.8%) completed the entire patient pathway and transition process (Fig. 5).

#### Duration of the transition process

The average time between the decision to include the patient in the transition process and the first appointment for training/counselling was 145.9 days (median 124; range 0—741 days). On average, 2.1 training sessions (median 2.0; range 1–5) were necessary and in about 95% of these an interdisciplinary team with a median of three participants (doctors, nurses, dieticians, psychologists) were involved of 357 days (median 350; range 0–819 days). A follow-up appointment was scheduled for all patients.

After an average of 352.2 days (SD 170.7; median 353; range 0–798) an interdisciplinary transfer clinic was conducted in 159 patients with the adult medicine consultant and in 109 patients with the paediatric team only. However, in both scenarios during this transfer appointment the structured epicrisis and the relevant accompanying documentation from the paediatric care was explained and delivered to the patient.

In 268 patients the transition process was completed after an average of 357 days (SD 180.6; median 350; range 0–819 days). A follow-up appointment was scheduled for all patients.

#### Referral to adult care

Eleven patients did not go through the entire transition process but nevertheless a health care provider in the adult care setting was identified for these patients. For 249 patients, disease-specific expert health care providers in adult care settings were identified. The majority of patients were transferred to a specialised disease-specific outpatient clinic in a university setting.

For 19 young adults it was not possible to identify a specialist in the adult health care setting. Of these, 12 remained in paediatric care. The remaining 7 patients were referred to adult medicine without disease-specific continuing care.

## Discussion

This is the first study evaluating a structured transition pathway in adolescent patients with any rare disease in a multi-centre setting. Of the 292 enrolled patients, 92.8% completed the process.

In 2002, American paediatric and adult societies jointly published a consensus statement, in which they demanded a structured transition process [23]. The goal of such a process is to support a person’s adjustment to a new care culture, to provide the necessary tools for selfcare and to help satisfy medical, psychosocial and educational needs [24]. However, when systematically evaluating the efficacy of a transition process, a major difficulty is the definition of ‘success’ [4]. Monitoring parameters and/or outcome parameters have been identified for either more common conditions (e.g. diabetes mellitus [25]) or for defined groups of diseases (e.g. kidney transplant [26]). For example, the HbA1c levels during and following the transition process have been used as a marker of glycaemic control in patients with diabetes mellitus. However, rare diseases affect only a few patients and an outcome or monitoring parameter would need to cover a wide variety of conditions.

We investigated the success of the transition process (a) by monitoring the completion rate of the different steps of the pathway (implementation rate) and (b) by measuring the health literacy in patients prior to entering the pathway and at completion of the pathway. At time of enrolment into the project, most patients had limited knowledge on disease-specific and general health-related topics. A structured education program was able to meet the needs of the patients and resulted in improved health literacy as measured by the questionnaire. Subsequently, 95.7% of the patients could be transferred to adult medical care and patients were satisfied with the transition pathway [18].

Unfortunately, the transition reality for patients with rare diseases in Germany is still insufficient, as outlined in a report by the German advisory council (Sachverständigenrat) in 2009 [27]. The report states that structured transition programs are lacking in Germany, except for some local solutions. Moreover, the report also recognizes a lack of sufficient financial compensation for the efforts associated with the transition of patients with rare diseases within the German health care system [27]. Since the publication of this report, no substantial structural changes in transition medicine or the funding of it have been implemented on a national level. With respect to RDs the European Reference Networks (ERN) may play an important role in transition medicine. Indeed, some ERNs have already published mappings of the current landscape like e.g. ENDO ERN [28], formed transition working groups e.g. ERN RITA [29] and provided recommendations and pathways for certain RDs [30].

In TRANSLATE NAMSE, patients were actively involved in the transition process by recording and identifying their training and counselling needs in the transition questionnaire at the beginning of the transition pathway. This led to improved patient empowerment through subsequent training and counselling [24, 31, 32]. Empowerment begins when the health care provider acknowledges that patients are in control of their daily care and when the health care provider’s goal is to increase the capacity of patients to think critically and make autonomous decisions [33]. Previous studies have shown that empowered adolescents can interact better with the adult health care system and are more independent and more actively involved in decisions regarding health [33, 34]. Thus, self-management improves health outcomes in RDs by improving adherence to the treatment plan and building the individual’s capacity to navigate challenges and solve problems.

The transition process in TRANSLATE NAMSE was complex and time-consuming. The average time from the decision to transition to the final consultation was around 1 year.

The individual process steps in the patient-oriented pathway were also time-consuming. With a median of 90 min, the first transition consultation and the transition consultation jointly with the adult specialist took the longest. The duration of the consultations ranged from 10 to 220 min [10].

In addition to the extended time that was required for the consultation clinics and that was mainly (but not exclusively) provided by the treating paediatricians, resources of other disciplines (e.g., administration for scheduling, occupational/nutritional therapists and other health care professions) were required in the process. A complete description of the resource consumption is available ([10]- in German only). The average total costs for the entire process were 599.00 € ± 380.50 €. These numbers are not transferable to other countries or health care systems but indicate that a structured and quality assured transition pathway can be achieved for a reasonable price.

There is widespread recognition that planning for transition and the transfer to adult care should be part of standard clinical care for all adolescents/young adults with chronic medical conditions [37]. The American Academy of Pediatrics (AAP) states that the referring paediatric providers must understand and address patients’ and parents’ perspectives and needs during transition [32]. With this in mind, and against the background of time- and cost- intensive patient pathways, there is a need for sustainable financial compensation especially in the paediatric sector, which is currently not adequately reflected in the remuneration. To lend weight to this demand, further studies on the costs of the transition process are needed [10]. These should especially take into account possible costs occurring without transition, e.g. treatment discontinuation.

## Limitations of the study

Funding by the German Federal Joint committee is given for projects in the German health care system with the purpose of improving health care provision to specific populations, such as patient with RD, through new interventions. As such, the project was limited to only 3 years and was not designed as a regular multi-centre study which would have included a control group. Thus, the two main limitations of this study are a lack of long-term follow-up data and the lack of a control group. In combination, this would have permitted an assessment of the effects of the adherence to medication and the medical system over a longer term. However, details regarding the key elements of the transition process as well as information pertaining to needs of patients facing transition to adult care could be provided. Nevertheless, it must be considered that inclusion in the study alone could be a confounder in answering the questionnaires presented.

It should also be noted that in the context of this study, the severity of the disease has not been accounted for. Thus, the transition process for patients who have a more severe condition could be significantly complicated and prolonged, as well as requiring more support and counseling. Furthermore, the availability of effective treatment is heterogeneous between different rare diseases. Nevertheless, this can have a lasting impact on the transition process. Thus, both issues need to be considered when planning and implementing the transition.

## Conclusion

This structured transition pathway is easily applicable for various rare diseases. The tools to assess patient knowledge are accessible via the questionnaire, the individualized training is offered by the highly specialised paediatrician and the transfer into adult care can be organized via a specific care coordinator. Thus, this patient pathway offers a universally applicable approach to adolescents with a RD. However, this transition pathway heavily relies on the necessary resources (time and personnel) in paediatric care settings and therefore sustainable funding needs to be established urgently.

## Supplementary Information

**Additional file 1. Supplementary table 1:** Calculation matrix to quantify training and counselling needs. Provided is the scoring system for the answers of the questionnaire. Information /counselling needs are expressed as % according to the answers of the patients. **Supplementary table 2:** List of diagnostic groups and diagnoses of patients who were enrolled in the study. **Supplementary Figure 1:** Flow Chart of the Transition Pathway. **Supplementary Figure 2:** Standardized and Structured Transition Questionnaire. **Supplementary Figure 3:** Excerpt from the "Patient Questionnaire on Transition” that was provided after completion of the study.

## Data Availability

Under the terms of the funding agency’s agreement data sharing, including related documents, is not permitted.

## Abbrevation

RD: Rare disease

## References

[1] Blum RWM, Garell D, Hodgman CH (1993). Transition from child-centered to adult health-care systems for adolescents with chronic conditions. J Adolesc Health.

[2] Sawyer SM, Drew S, Yeo MS, Britto MT (2007). Adolescents with a chronic condition: challenges living, challenges treating. Lancet.

[3] Kashikar-Zuck S (2021). Transition of care for adolescents with chronic pain. Lancet Child Adolesc Health.

[4] Kordonouri O (2017). Transition of care for young adults with chronic diseases. Lancet Child Adolesc Health.

[5] Hölling H, Schlack R, Kamtsiuris P, Butschalowsky H, Schlaud M, Kurth BM (2012). The KiGGS study: nationwide representative longitudinal and cross-sectional study on the health of children and adolescents within the framework of health monitoring at the Robert Koch Institute. Bundesgesundheitsblatt—Gesundheitsforschung—Gesundheitsschutz.

[6] van Walleghem N, MacDonald CA, Dean HJ (2008). Evaluation of a systems navigator model for transition from pediatric to adult care for young adults with type 1 diabetes. Diabetes Care.

[7] Schütz L, Radke M, Menzel S, Däbritz J (2019). Long-term implications of structured transition of adolescents with inflammatory bowel disease into adult health care: A retrospective study. BMC Gastroenterol.

[8] Watson AR (2000). Non-compliance and transfer from paediatric to adult transplant unit. Pediatr Nephrol.

[9] Rettig P, Athreya BH (1991). Adolescents with chronic disease. Transition to adult health care. Arthritis Care Res: Off J Arthritis Health Prof Assoc.

[10] Grasemann C, Höppner J, Burgard P (2021). Resource use of structured transition of young people with rare disease from pediatrics to adult medicine. Monatsschrift Kinderheilkunde.

[11] Nabbout R, Arzimanoglou A, Chin RFM, Grinspan Z, Speechley K, Camfield P (2019). The evaluation and costs of transition programs for youth with epilepsy. Epilepsy Behav.

[12] Bavisetty S, Grody WW, Yazdani S (2013). Emergence of pediatric rare diseases. Rare Diseases.

[13] Royal College of Nursing. *Adolescent Transition Care*.; 2013:1–25.

[14] DH/Child Health and Maternity Services Branch. Transition: getting it right for young people Improving the transition of young people with long term conditions from children’s to adult health services. Published online 2006.

[15] Oppong-Odiseng ACK, Heycock EG (1997). Adolescent health services: through their eyes. Arch Dis Child.

[16] Brooks F, Bunn F, Morgan J (2009). Transition for adolescents with long-term conditions: event to process. Br J Commun Nurs.

[17] Clarizia NA, Chahal N, Manlhiot C, Kilburn J, Redington AN, McCrindle BW (2009). Transition to adult health care for adolescents and young adults with congenital heart disease: perspectives of the patient, parent and health care provider. Can J Cardiol.

[18] Agency for Clinical Innovation and Trapeze, The Sidney Children’s Hospitals Network. Key principles for transition of young people from paediatric to adult health care. 2014. https://www.aci.health.nsw.gov.au/__data/assets/pdf_file/0011/251696/Key_Principles_for_Transition.pdf

[19] Grasemann C, Matar N, Bauer J (2020). Development of a structured transition program for adolescents and young adults with a chronic rare disease: results from the German consortium TRANSLATE NAMSE. Monatsschrift fur Kinderheilkunde.

[20] Rillig F, Grüters A, Bäumer T (2022). Diagnosis of rare diseases-results of the TRANSLATE NAMSE project. Deutsches Arzteblatt Int.

[21] Choukair D, Lee-Kirsch MA, Berner R (2022). The clinical pathway for multidisciplinary treatment of rare diseases in paediatrics: results from the TRANSLATE NAMSE project. Monatsschrift fur Kinderheilkunde.

[22] TRANSLATE NAMSE. Improving Care for People with Rare Diseases through Implementation of Measures Consented in the National Action Plan (NAMSE)—G-BA Innovation Fund. https://innovationsfonds.g-ba.de/beschluesse/translate-namse-verbesserung-der-versorgung-von-menschen-mit-seltenen-erkrankungen-durch-umsetzung-von-im-nationalen-aktionsplan-namse-konsentierten-massnahmen.66. Accessed April 5, 2022

[23] American Academy of Pediatrics, American Academy of Family Physicians (2002). American college of physicians-American society of internal medicine. A consensus statement on health care transitions for young adults with special health care needs. Pediatrics.

[24] Acuña Mora M, Sparud-Lundin C, Bratt EL, Moons P (2017). Person-centred transition programme to empower adolescents with congenital heart disease in the transition to adulthood: a study protocol for a hybrid randomised controlled trial (STEPSTONES project). BMJ Open.

[25] White M, O’Connell MA, Cameron FJ (2017). Clinic attendance and disengagement of young adults with type 1 diabetes after transition of care from paediatric to adult services (TrACeD): a randomised, open-label, controlled trial. Lancet Child Adolesc Health.

[26] Prüfe J, DIerks ML, Bethe D, (2017). Transition structures and timing of transfer from paediatric to adult-based care after kidney transplantation in Germany. A qualitative study. BMJ Open.

[27] Council of Experts on the Assessment of Developments in the Health Care System. *Coordination and Integration: Health Care in a Society of Longer Life*.; 2009. 10.5771/9783845221687

[28] Acuña Mora M, Moons P, Sparud-Lundin C, Bratt EL, Goossens E (2016). Assessing the level of evidence on transfer and transition in young people with chronic conditions: protocol of a scoping review. Syst Rev.

[29] Cooley WC, Sagerman PJ, Barr MS (2011). Supporting the health care transition from adolescence to adulthood in the medical home. Pediatrics.

[30] Anderson RM, Funnell MM (2010). Patient empowerment: myths and misconceptions. Patient Educ Couns.

[31] Bravo P, Edwards A, Barr PJ, Scholl I, Elwyn G, McAllister M (2015). Conceptualising patient empowerment: a mixed methods study. BMC Health Serv Res.

[32] Small N, Bower P, Chew-Graham CA, Whalley D, Protheroe J (2013). Patient empowerment in long-term conditions: development and preliminary testing of a new measure. BMC Health Serv Res.

[33] Lozano P, Houtrow A (2018). Supporting self-management in children and adolescents with complex chronic conditions. Pediatrics.

[34] Maddux MH, Ricks S, Bass J (2017). Patient and Caregiver perspectives on transition and transfer. Clin Pediatr.

