# Supplementary material for: Transition for adolescents with a rare disease: results of a nationwide German project
Source: Orphanet J Rare Dis. 2023 Apr 25;18:93. doi: 10.1186/s13023-023-02698-2 (PMC10131406; doi:10.1186/s13023-023-02698-2)
Supplement: Supplementary file 1 — Additional file 1. Supplementary table 1: Calculation matrix to quantify training and counselling needs. Provided is the scoring system for the answers of the questionnaire. Information /counselling needs are expressed as % according to the answers of the patients. Supplementary table 2: List of diagnostic groups and diagnoses of patients who were enrolled in the study. Supplementary Figure 1: Flow Chart of the Transition Pathway. Supplementary Figure 2: Standardized and Structured Transition Questionnaire. Supplementary Figure 3: Excerpt from the "Patient Questionnaire on Transition” that was provided after completion of the study. [file 13023_2023_2698_MOESM1_ESM.docx]

**Supplementary Material**

***Supplementary Tables and Figures***

| **Supp. Tab. 1: Calculation matrix to quantify training and counselling needs**  The matrix provides the calculation for the quantification of training and counselling needs. | | | | | |
| --- | --- | --- | --- | --- | --- |
| **Surveyed Domains** | **Question from the transition questionnaire**  (supp. Fig. 2) | **Answer from transition questionnaire** (supp. Fig. 2) | | | |
|  |  | Agree (%) | partially Agree (%) | Disagree (%) | Does not apply to my condition |
| Disease specific | I know the name of my condition and can explain it to others. | 0 | 8.33 | 16.66 | 0 |
|  | I understand which examinations and tests are being done and why. | 0 | 8.33 | 16.66 | 0 |
|  | I know which symptoms and complications can occur in the course of my condition. | 0 | 8.33 | 16.66 | 0 |
|  | I recognize signs of deterioration of my condition. | 0 | 8.33 | 16.66 | 0 |
|  | I am familiar with the effects of my condition/treatment in the event of pregnancy or childbirth. | 0 | 8.33 | 16.66 | 0 |
|  | I would like the treatment team to provide more information about my illness. | 0 | 8.33 | 16.66 | 0 |
| **Sum Disease specific (%)** | | **0** | **50** | **100** |  |

| Therapy related | I am familiar with the different aspects of my treatment. | 0 | 6.25 | 12.5 | 0 |
| --- | --- | --- | --- | --- | --- |
|  | I know the names of my medication. | 0 | 6.25 | 12.5 | 0 |
|  | I am familiar with the effects and side effects of my treatment/medication. | 0 | 6.25 | 12.5 | 0 |
|  | I take my medication independently, and/or carry out my treatment independently. | 0 | 6.25 | 12.5 | 0 |
|  | I take my medication regularly, and/or carry out my treatment regularly. | 0 | 6.25 | 12.5 | 0 |
|  | I can adapt my medication/treatment as necessary to certain circumstances (e.g. stress, infection, leisure time). | 0 | 6.25 | 12.5 | 0 |
|  | I know which factors may influence the effect of my medication. | 0 | 6.25 | 12.5 | 0 |
|  | I am familiar with necessary adjustments and information in the event of a surgical procedure. | 0 | 6.25 | 12.5 | 0 |
| **Sum Therapy related (%)** | | **0** | **50** | **100** |  |

| Medical System | If necessary: I have an emergency ID and carry it with me. | 0 | 5.55 | 11.11 | 0 |
| --- | --- | --- | --- | --- | --- |
|  | I receive sufficient support from my partner, my parents, or other adult confidants. | 0 | 5.55 | 11.11 | 0 |
|  | I am close to an adult (friend- or family) who is well informed about my condition and its treatment. | 0 | 5.55 | 11.11 | 0 |
|  | I feel adequately informed about support offers. | 0 | 5.55 | 11.11 | 0 |
|  | I know how to get in contact with other people who have the same condition. | 0 | 5.55 | 11.11 | 0 |
|  | During clinic appointments I speak to my doctor alone, or choose who accompanies me. | 0 | 5.55 | 11.11 | 0 |
|  | I can arrange my clinic appointments by myself. | 0 | 5.55 | 11.11 | 0 |
|  | I know how to contact my treatment team (e.g. doctors, diet consultants). | 0 | 5.55 | 11.11 | 0 |
|  | I can take order prescriptions/refills myself. | 0 | 5.55 | 11.11 | 0 |
| **Sum Medical System (%)** | | **0** | **50** | **100** |  |

| Lifestyle | I am familiar with potential consequences of my condition on sexuality and contraception. | 0 | 7.14 | 14.29 | 0 |
| --- | --- | --- | --- | --- | --- |
|  | I am familiar with the potential impact of tobacco, alcohol, and other drugs on my condition and treatment. | 0 | 7.14 | 14.29 | 0 |
|  | I know what to consider when preparing for travel. | 0 | 14.29 | 14.29 | 0 |
|  | I am familiar with potential effects of my condition/treatment on my ability to obtain a drivers license and drive a vehicle. | 0 | 7.14 | 14.29 | 0 |
|  | I know what my teacher/employer needs to know about my condition. | 0 | 7.14 | 14.29 | 0 |
|  | I understand the potential impact of my condition on my ability to work and take that into consideration when choosing a job. | 0 | 7.14 | 14.29 | 0 |
|  | I would like the treatment team to provide support with questions about school, training, studies, and work. | 14.29 |  | 0 | 0 |
| **Sum Lifestyle (%)** | | **14.3** | **50** | **85.7** |  |

| Psychological counselling | I would like the treatment team to provide psychological counselling. | 100 |  | 0 | 0 |
| --- | --- | --- | --- | --- | --- |
| **Sum Psychological counselling (%)** | | 100 |  | 0 |  |

| Socio-legal counselling | My condition or its treatment is a financial burden for me. | 50 | 25 | 0 | 0 |
| --- | --- | --- | --- | --- | --- |
|  | I would like the treatment team to provide information on how to deal with authorities and legal changes after reaching the majority age of 18 years (e.g. health insurance, administrations, insurance). | 50 |  | 0 | 0 |
| **Sum Socio-legal counselling** | | **100** | **25** | **0** |  |

| Genetic counselling | I understand the underlying genetic/inheritance rules of my condition. | 0 | 50 | 100 | 0 |
| --- | --- | --- | --- | --- | --- |
| **Sum Genetic counselling (%)** | | 0 | 50 | 100 |  |
| Each surveyed domain was evaluated separately. A need of 0% was evaluated as no need for training or counseling, a need <20% as medium, and a need >20% as high. | | | | | |

**Supplementary Tables**

| **Supp. Table 2: List of diagnostic groups and diagnoses** | | | | | |
| --- | --- | --- | --- | --- | --- |
| **Hematologic** | **Endocrine** | **Autoimmune** | **Metabolic** | **Nephrologic** | **Others** |
| Acute lymphatic leukemia | Vitamin D Hydroxylation-deficient rickety, Type 1A | Rare autoimmune disease | 3-Methylcrotonyl-CoA-Carboxylase Deficiency | ANCA-Vasculitis | Chronic myeloid leukemia (CML) |
| Aplastic anaemia | 4H Syndrome | Lyme Disease | Citrullinaemia Type 1 | Cystinosis | T-Cell-Lymphoma |
| Blackfan-Diamond anemia | Addison’s Disease | Autoinflammatory disease | Cobalamin A-Deficiency | Autosomal dominant polycystic kidney disease | Syndromic developmental delay |
| Haemochromatosis | Diabetes insipidus centralis | Vasculitis | Hawkinsinuria | Bartter-Syndrome Type III | Duchenne muscular dystrophy |
| Iron refractory iron deficiency anemia | Adrenal Gland Adenoma | Juvenile idiopathic arthritis (JIA) | Isovaleric acidaemia | CAKUT | Encephalopathy (TRIM8) |
| Thalassaemia | Congenital adrenal hyperplasia (CAH) | Autoimmune thrombocytopenia | Isovaleric aciduria | Multicyclic dysplastic kidney | Mb. Hodgkin |
|  | Disorders of parathyroid hormone signaling; Pseudohypoparathyroidism Type 1 A | Chronic recurrent multi-focal osteomyelitis | Medium-Chain-Acyl-CoA-Dehydrogenase-Deficiency | Atypical haemolytic uraemic Syndrome (aHUS) | Multifocal Langerhans cell histiocytosis |
|  | Growth hormone secreting pituitary adenoma | Familial Mediterranean fever (FMF) | Methylmalonic acidemia | Nephrotic syndrome, steroid resistant | Primary sclerosing cholangitis (PSC) |
|  | Congenital adrenal Gland Hyperplasia due to 21-Hydroxylase deficiency | Systemic Lupus Erythematosus (SLE) | Methylmalonic aciduria | EHEC HUS | Neuroblastoma |
|  | Anorchia | Sacroiliitis | Phenylketonuria (PKU) | Galloway-Mowat-Syndrome | Neurofibromatosis |
|  | APECED-Syndrome |  |  | Hyperoxaluria | Septic granulomatosis |
|  | Cushing’s Disease |  |  | Hypodysplastic kidney |  |
|  | Short Stature (Laron Syndrome) |  |  | Idiopathic Hypercalciuria |  |
|  | Hypothyroidism |  |  | IgA Glomerulonephritis |  |
|  | Graves Disease |  |  | Lowe-Syndrome |  |
|  | Hypoparathyroidism sensorineural deafness and renal dysplasia syndrome |  |  | Nephrotic syndrome, steroid responsive |  |
|  | Hereditary Thyroid carcinoma |  |  | Nephronophthisis |  |
|  | Disorders of sexual development |  |  | Polyangiitis |  |
|  | Hypergonadotropic Hypogonadism |  |  | Posterior urethral valves |  |
|  | Hypogonadotropic Hypogonadism |  |  | Prune-Belly-Syndrome |  |
|  | Hypoparathyroidism |  |  | Dent's Disease Type II |  |
|  | (Pan)-Hypopituitarism |  |  | Mainzer-Saldino Syndrome |  |
|  | Juvenile Osteoporosis |  |  | Tubulointerstitial nephritis |  |
|  | Kallmann-Syndrome |  |  |  |  |
|  | Craniopharyngioma with panhypopituitarism |  |  |  |  |
|  | McCune Albright Syndrome |  |  |  |  |
|  | MEN 1 |  |  |  |  |
|  | MEN 2a |  |  |  |  |
|  | Microdeletion 22q11.2 |  |  |  |  |
|  | Noonan Syndrome |  |  |  |  |
|  | Prader-Willi-Syndrome |  |  |  |  |
|  | Prolactinoma |  |  |  |  |
|  | Thyroid cancer |  |  |  |  |
|  | Thyroid nodule |  |  |  |  |
|  | Transgender |  |  |  |  |
|  | Ullrich Turner Syndrome |  |  |  |  |
|  | Androgen-insensitivity Syndrome |  |  |  |  |

**Supplemental Figures**

**Supp. Fig. 1: Flow Chart of the Transition Pathway**


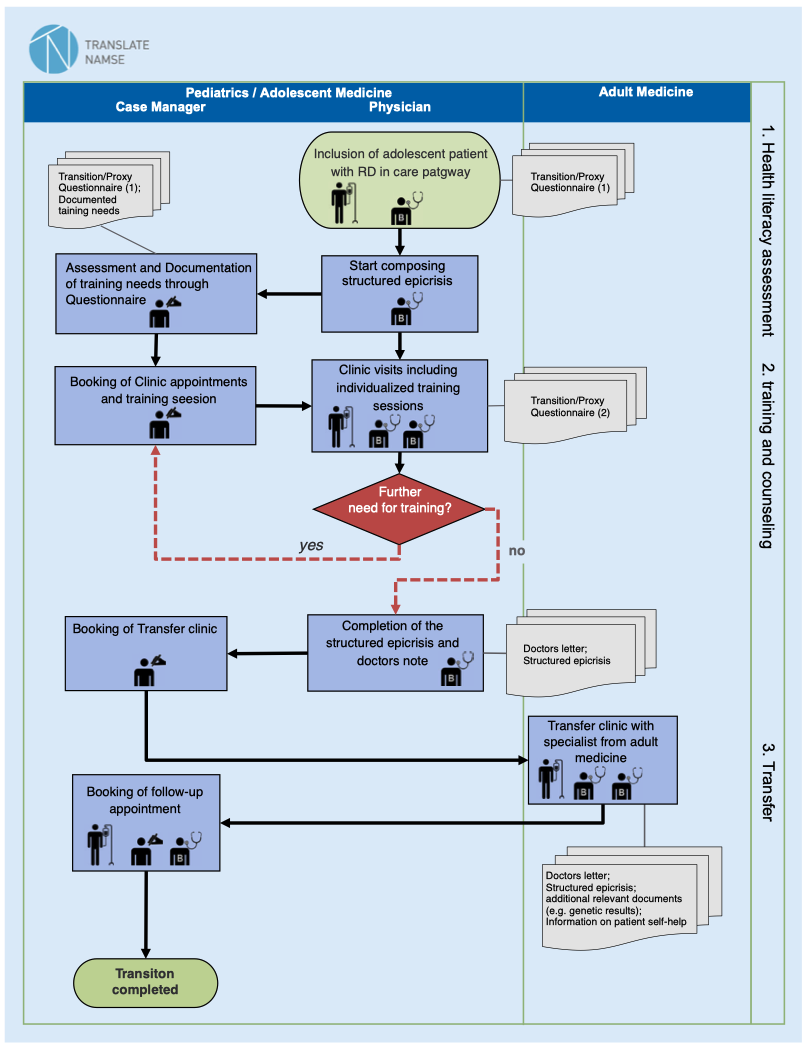


**Supp. Fig. 2: Standardized Transition Questionnaire**

**
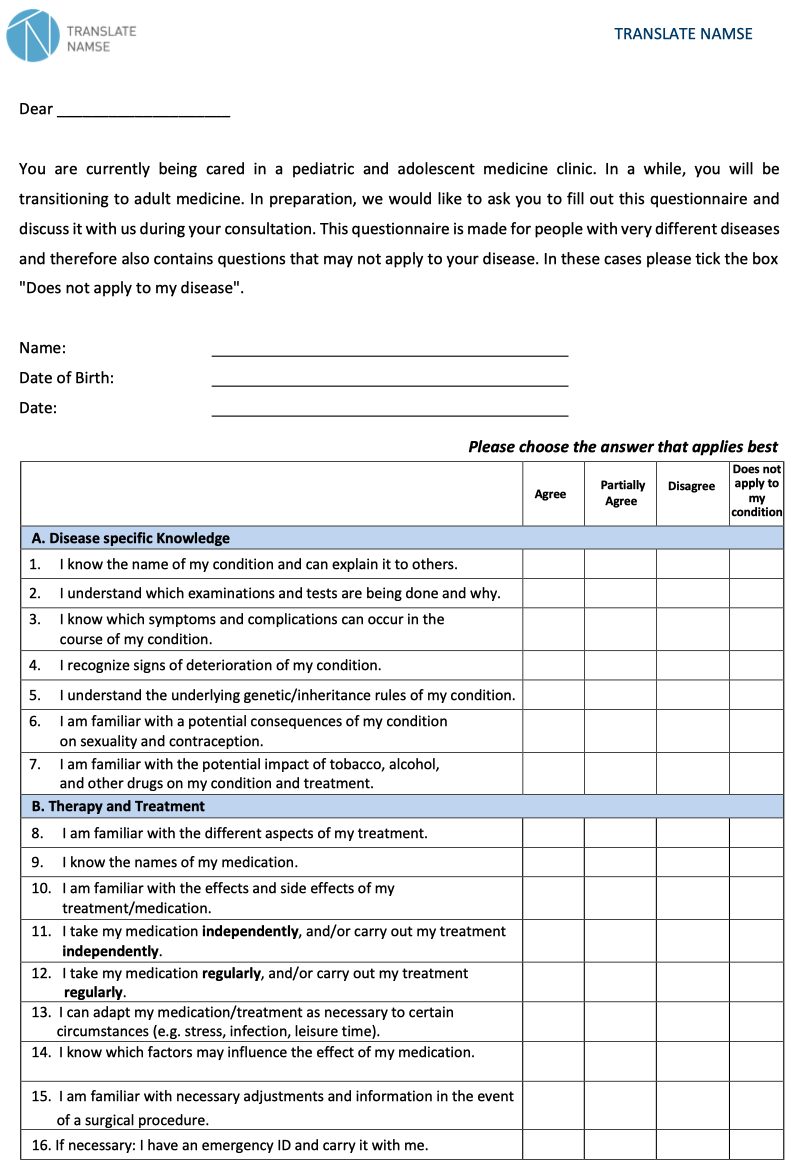
**

**
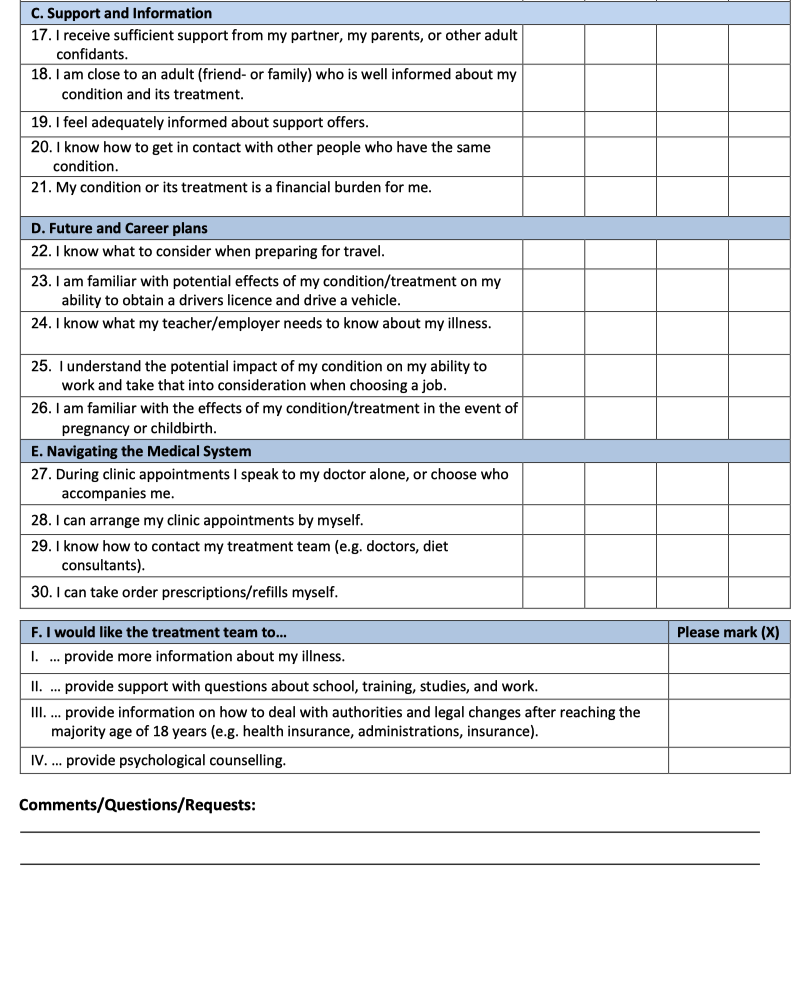
**

**Supp. Fig. 3: Excerpt from the "Patient Questionnaire on Transition”**

**
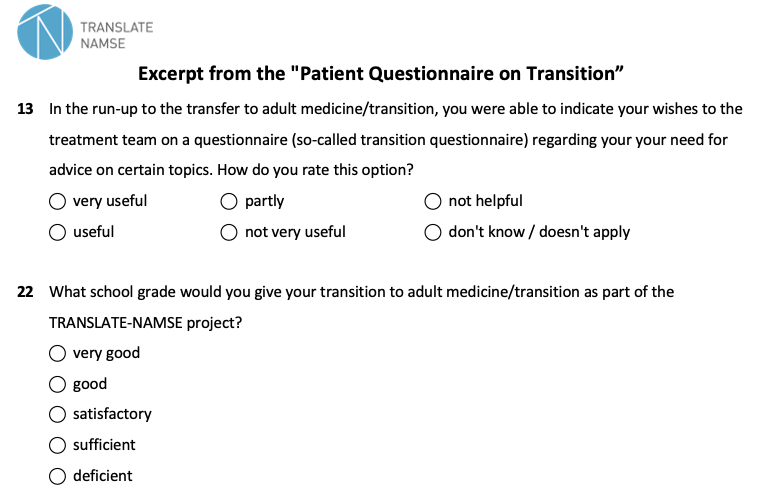
**
